# Inhibition of L-threonine dehydrogenase from *Trypanosoma cruzi* reduces glycine and acetate production and interferes with parasite growth and viability

**DOI:** 10.1016/j.jbc.2024.108080

**Published:** 2024-12-13

**Authors:** Jessica do Nascimento Faria, Amanda G. Eufrásio, Michelle Fagundes, Angel Lobo-Rojas, Letícia Marchese, Caio Cesar de Lima Silva, Eduardo H.S. Bezerra, Gustavo F. Mercaldi, Marcos R. Alborghetti, Mauricio L. Sforca, Artur T. Cordeiro

**Affiliations:** 1 Brazilian Biosciences National Laboratory, Brazilian Center for Research in Energy and Materials, Sao Paulo, Brazil; 2 Faculty of Pharmaceutic Sciences, University of Campinas, Sao Paulo, Brazil

**Keywords:** Chagas Box, target identification, potassium binding, metabolism, substrate-level phosphorylation, drug discovery

## Abstract

*Trypanosoma cruzi* is a flagellated protozoan and the etiological agent of Chagas disease, a neglected tropical disease described by Carlos Chagas in 1909 that remains without appropriate diagnostics and treatment. Throughout its life cycle, *T*. *cruzi* undergoes through many different environments, requiring adaptation of its metabolism to different nutrition sources. Recent studies have confirmed the adaptability of *T*. *cruzi* metabolism to different carbon sources and encouraged a deeper investigation of related metabolic pathways. In the present study, we investigated the catabolism of threonine in *T*. *cruzi* epimastigotes cultivated in LIT medium and following 24h of starvation in PBS. In LIT medium, threonine, serine, and histidine were rapidly consumed concomitantly with carbohydrates during parasite exponential growth. When threonine was provided as the only carbon source to starved parasites, they excreted acetate and glycine, corroborating the activity of a mitochondrial threonine degradation pathway. Subsequently, we used a recombinant *T*. *cruzi* L-threonine dehydrogrenase (TcTDH) to screen the Chagas Box, an open-source collection of phenotypic hits, and identified compound TCMDC-143160 as a low micromolar TcTDH inhibitor (IC50 = 3.5 μM). When TCMDC-143160 was administrated to starved parasites, it inhibited the threonine degradation pathway. Finally, we report the crystal structure of TcTDH and characterize its allosteric activation by potassium. Collectively, these data demonstrate the relevance of threonine catabolism in *T*. *cruzi* metabolism and provide a set of tools to further investigate TcTDH as a potential drug target for Chagas disease.

The flagellated protozoan *Trypanosoma cruzi* is the etiological agent of Chagas disease (CD), a neglected tropical disease, endemic in 21 countries, where six to seven million people are currently infected and other 75 million lives in areas of considerable risk of infection (1). During its life cycle, different forms of the parasite can be found in the digestive tube of blood feeding insects, in the bloodstream, and in the cytosol of mammalian host cells, usually from heart, esophagus, and colon tissues. Living in such different environments requires the ability to adapt its metabolism to different nutrition sources. Recent metabolomic studies confirm *T*. *cruzi* adaptability to different carbon sources and encourage a deeper investigation of related metabolic pathways (2).

*Trypanosoma brucei* procyclic forms consume and metabolize threonine to acetate, which is used for lipids biosynthesis (3). In this parasite, threonine degradation pathway occurs within the mitochondrion, where the amino acid is initially oxidized to 2-amino-3-ketobutyrate (AKB) by a NAD^+^ dependent L-threonine dehydrogenase (TDH, 1.1.1.103) and then cleaved into glycine and acetyl-CoA by a 2-amino-3-ketobutyrate coenzyme A ligase (AKCL, 2.3.1.29). The produced acetyl-CoA will be converted to acetate through the action of either acetate:succinate CoA-transferase (ASCT, EC 2.8.3.8) or acetyl-CoA thioesterase (ACH, EC 3.1.2.1) (4). Acetate and glycine are the main end-products of the catabolism of threonine and both are secreted at equal amounts to the extracellular medium (5). In *T*. *cruzi*, except for ACH, all enzymes required for the degradation of threonine have putative genes annotated in TriTrypsDB, so presumably, these pathways should also be functional in this parasite.

TDHs from distinct species vary in quaternary structure and mechanistic properties. In general, dehydrogenases are classified into short- (SDR), medium- (MDR), or long-chain (LDR) families based on their length and structural organization. Medium-chain TDHs have been characterized for *Pyrococcus horikoshii* (6) and *Thermococcus kodakaraensis* (7). These enzymes fold as tetramers with a Rossmann-fold domain, for cofactor binding, at the C-terminal of each monomer and at least one metal binding site per chain, frequently occupied by zinc. Crystal structures of short-chain TDHs are available for *Flavobacterium frigidimaris* (8), *Thermoplasma volcanium* (9), *Cupriavidus necator* (CnTDH) (10), mouse (11), and *T*. *brucei* (12). Their tertiary structures are similar to UDP-galactose four-epimerase. Both enzymes form homodimers with the Rossman-fold domain at the N-terminus and two conserved sequence signatures: GxxGxxG, which is involved in cofactor binding and YxxxK, with the tyrosine residue participating in catalysis mechanism. The reaction mechanism of SDR-TDHs was precisely described using the tridimensional enzyme structure from *T*. *volcanium* (9). It starts with the proton abstraction from the β-hydroxyl of the threonine side chain, mediated by the conserved tyrosine residue. Then, a proton from β-carbon in the reaction intermediate is transferred to NAD^+^, resulting in the formation of AKB and NADH. In the mouse homologous enzyme (mTDH), a “remote switch region” was proposed to control the enzyme activity (11). Mutation or methylation of Arg-180, localized in this region, causes a reduction or increase in mTDH activity, respectively (13). In mouse embryonic stem cells (mESC), threonine catabolism is essential for cellular growth and differentiation (14). Indeed, inhibition of mTDH by a carboxiquinazoline (Qc1) arrests growth of mESC, but not of human HeLa cells (15). This selectivity is attributed to the absence of a functional TDH in human cells, which is coded by a pseudo gene, and its expression results in a truncated polypeptide chain. The absence of TDH in humans makes it an attractive drug target for infectious diseases caused by parasites, which depend on the threonine degradation pathways for growth and survival.

Given the absence of TDH in humans and the importance of threonine for the differentiation of totipotent cells, we sought to investigate the relevance of threonine catabolism for *T*. *cruzi* growth and to assess whether *T*. *cruzi* TDH (TcTDH) could be explored as a potential target in future drug discovery initiatives against Chagas disease. In the present work, we (i) utilized ^1^H-NMR to assess threonine degradation pathway in *T*. *cruzi*, (ii) identified a novel inhibitor for TcTDH that arrests the growth of both epimastigote and amastigote forms, (iii) demonstrated its activity on threonine degradation within the parasite, and (iv) elucidated new aspects of TcTDH structure and function, such as its allosteric activation by potassium ion. Notably, TDH activation by potassium was first described by Margaret Green in the 1960s, in partially purified TDH from *Staphylococcus aureus* (16). However, to the best of our knowledge, this property has either been overlooked or is not relevant to other recently investigated TDHs. In conclusion, we demonstrated the importance of threonine degradation for *T*. *cruzi* growth and identified TCMDC-143160 as a novel chemical tool for studying the catabolism of threonine in trypanosomes.

## Results

### Threonine, serine, and histidine are promptly consumed by *T*. *cruzi* growing in LIT medium

**Figure 1:**
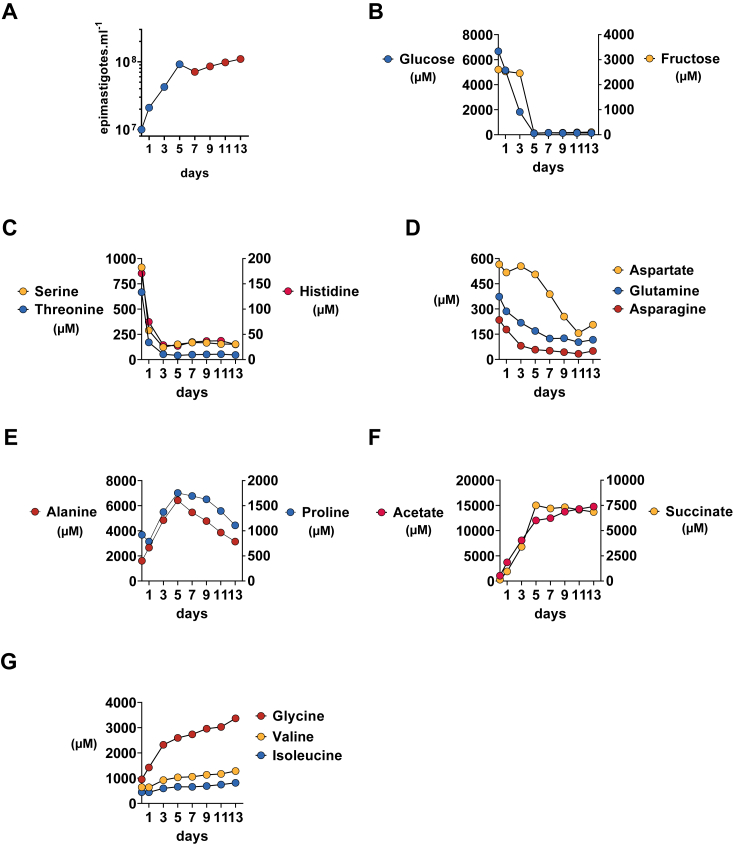
**Exometabolomics of *T*. *cruzi* epimastigotes growing in LIT medium for 13 days**. *A*, growth curve of *T*. *cruzi* epimastigotes in LIT medium. Exponential and stationary phases are colored blue and red, respectively. *B*–*D*, carbohydrates and amino acids consumed during parasite growth. *E*, amino acids excreted during the exponential growth phase and consumed in the stationary phase. *F*, main end-products of *T*. *cruzi* metabolism. *G*, other amino acids whose concentration increase over time. Dots represent the means of three technical replicates prepared from the same culture flask at distinc time points.

To investigate the consumption of threonine by *T*. *cruzi*, we utilized ^1^H-NMR spectroscopy to analyze the constituents of the LIT medium used for parasite growth over a 13-days period. Fifty-four metabolites were identified using an untargeted approach (Figs. S1 and 2). During the first 5 days, the parasite culture exhibited a doubling time of approximately 2 days until it entered a stationary phase (Fig, 1*A*). Glucose and fructose were identified as the most abundant carbohydrates in LIT medium, and parasites consume them entirely during exponential growth within 5 days (Fig. 1*B*). Glucose was observed to be the preferred carbohydrate source, as consumption of fructose only commenced after the third day, when the concentration of glucose decreased to approximately 2 mM. Total carbohydrate depletion at day 5 coincided with the transition from the exponential to the stationary growth phase.

Among the amino acids available in the LIT medium, threonine, serine, and histidine were rapidly consumed in the first 3 days (Fig. 1*C*), concomitantly with carbohydrates. Glutamine and asparagine consumption commenced on the first day, although at slower rates, while aspartate consumption occurred only in the stationary phase (Fig. 1*D*). Proline and alanine were synthesized and secreted during exponential growth and consumed in the stationary phase (Fig. 1*E*), when epimastigotes began to differentiate into metacyclic trypomastigotes. Acetate and succinate were the major end-products of *T*. *cruzi* metabolism, growing in LIT medium (Fig. 1*F*). Succinate was excreted exclusively during the exponential growth phase, whereas acetate excretion was observed at the entire experiment, albeit at a reduced rate in the stationary phase. Valine, isoleucine, and glycine were also excreted throughout the 13 days of the experiment (Fig. 1*G*). However, glycine secretion was considerably higher in the first 3 days of parasite exponential growth, and it showed an inverse relationship with threonine uptake.

### Catabolism of threonine occurs *via* the TDH-mediated pathway

**Figure 2:**
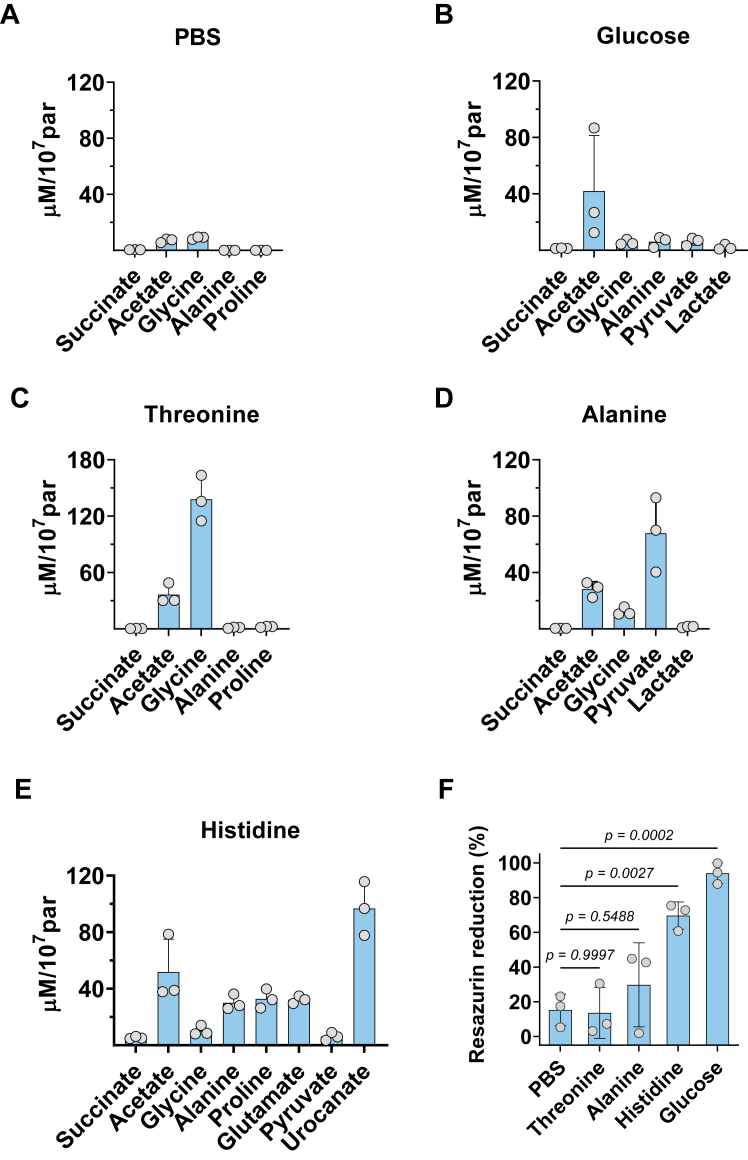
**Metabolites secreted by *T*. *cruzi* under nutrient restriction conditions**. 1 × 10^7^ parasites growing in LIT medium were submitted to 24h starvation in PBS and then rescued with 5 mM of different nutrients (*A*–*E*). After additional 24h, metabolites in the extracellular medium were quantified by ^1^H-NMR and the parasite redox state evaluated by resazurin reduction assay (*F*). Multiple comparisons of normalized resazurin reduction means of distinct nutrients treatment *versus* PBS were conducted by ordinary one-way ANOVA followed by Dunnett's test for significance (*p* < 0.05 for a significant difference). Bars and lines indicate the means and SD of three independent experiments.

The elevated glycine production during the first 3 days of parasite growth suggests a TDH-dependent threonine degradation pathway. To examine this hypothesis, we investigated the metabolism of parasites subjected to a 24h starvation period in PBS following the addition of various nutrients: threonine, histidine, alanine, and glucose. Moreover, we also evaluated the ability of such nutrients to produce reducing equivalents *via* a resazurin reduction assay. Glucose and histidine degradation are known to restore the reducing equivalent levels, similar to LIT medium, but not alanine. Starved parasites maintained in PBS for additional 24h ceased the excretion of most metabolites, leaving only traces of acetate and glycine in the extracellular medium (Fig. 2*A*). Acetate was excreted under all tested conditions, and it was the sole end-product of glucose catabolism (Fig. 2*B*). Parasites treated with threonine excreted higher levels of glycine compared to acetate (Fig. 2*C*). Such increase in glycine excretion was observed exclusively for parasites under this condition. The catabolism of alanine produced acetate and pyruvate (Fig. 2*D*), whereas glycine levels remained comparable to parasites in the control group maintained in PBS. Finally, the catabolism of histidine resulted in excretion of acetate, alanine, proline, glutamate, and urocanate but not glycine (Fig. 2*E*). These results suggest that increased glycine levels could serve as a marker for the activity of a TDH-dependent threonine degradation pathway in *T*. *cruzi*.

The threonine degradation pathway will also generate acetyl-CoA in the parasite mitochondrion. Acetyl-CoA can be further oxidized to CO_2_ *via* the Krebs Cycle or converted to acetate *via* the Acetyl-CoA succinate transferase (ACST)/Succinyl-CoA synthetase (SCoAS) cycle. The flux of metabolites through the Krebs Cycle must increase the level of reducing equivalents in the parasite mitochondrion and, consequently, an elevated signal in viability assays based on resazurin reduction. Thus, to assess whether the acetyl-CoA derived from threonine would follow the Krebs Cycle or the ACST/SCoAS cycle, we measured the resazurin reduction by starved parasites rescued with different carbon sources (Fig. 2*F*). Threonine and alanine did not increase the reduction of resazurin compared to parasites maintained in PBS. This result suggests that under such conditions, acetyl-CoA derived from threonine or from alanine is mainly converted into acetate by the ACST/SCoAS cycle.

### The inhibition of TDH interferes with the catabolism of threonine in *T*. *cruzi* epimastigotes

**Figure 3:**
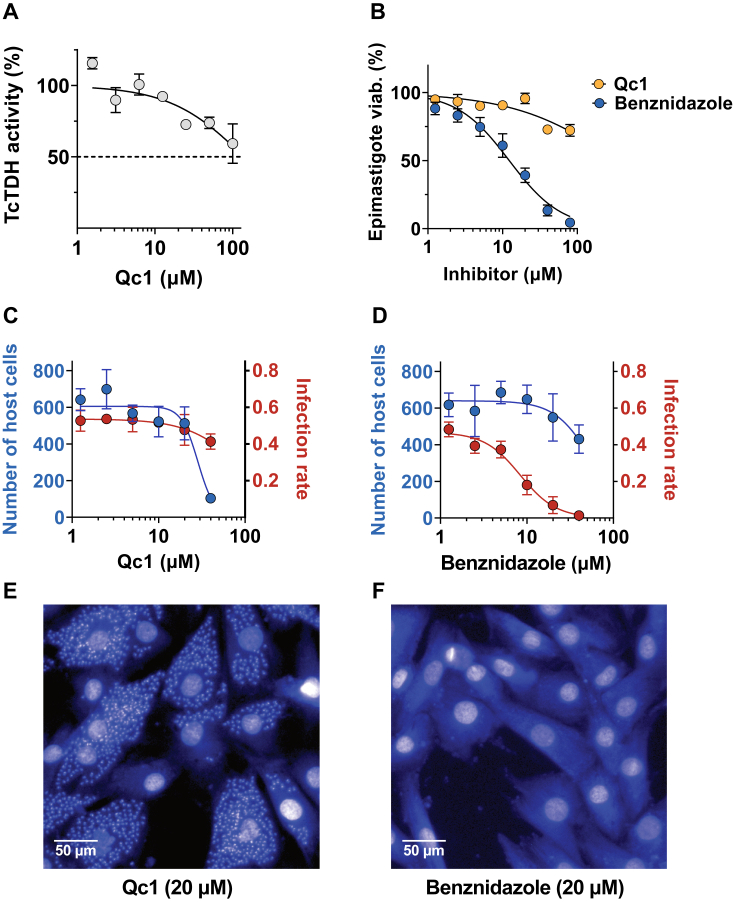
**Effect of Qc1 on TcTDH enzymatic activity and *in vitro* parasite growth**. *A*, Qc1 inhibition of TcTDH enzymatic activity. *B*, Comparison of Qc1 and Benznidazole, the reference drug for Chagas disease, on *T*. *cruzi* epimastigotes viability assay. Effect of Qc1 (*C*) and Benznidazole (*D*) on *T*. *cruzi* intracellular image-based assay. *Blue* dots represent the total number of host cells (H9c2 rat cardiomyocytes) imaged per well, while red dots account for the infection rate, calculated as the ratio of cells containing at least three amastigotes in the cytoplasm area. Representative images of *T*. *cruzi* infected host cells, after 72h treatment with Qc1 (*E*) and Benznidazole (*F*), both at 20 μM. Intracellular parasites are observed as small dots around the larger host nuclei. All the experiments were performed in biological triplicate.

To evaluate the relevance of the threonine degradation pathway in *T*. *cruzi* growth we tested the commercially available inhibitor of mTDH designated Qc1 (IC50 = 0.5 μM), which selectively inhibits the growth of mouse embryonic stem cells (EC50 = 3 μM), for its potential inhibition against TcTDH (15). However, our results indicated Qc1 is not a good inhibitor for the recombinant TcTDH, with IC50 > 100 μM (Fig. 3*A*). Furthermore, in comparison to benznidazole, the reference drug for Chagas disease, Qc1 had no effect on *T*. *cruzi* epimastigote viability (Fig. 3*B*), neither on the growth of intracellular amastigotes (Fig. 3, *C*–*F*). The poor inhibition of TcTDH by Qc1 may be attributed to structural differences between parasite and mouse TDHs, as their primary sequences share only 42% identity.

**Figure 4:**
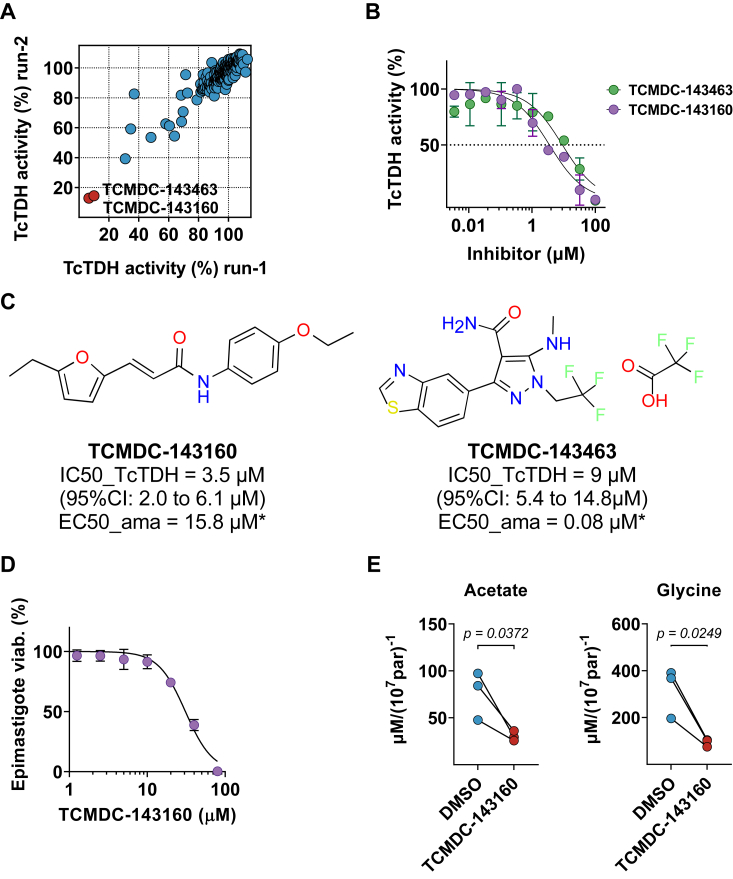
**Screening of ChagasBox for inhibitors of *T*. *cruzi* TDH**. *A*, correlation plot between two screening assays of Chagas Box compounds against TcTDH activity. In both runs, compounds were assayed at 10 μM, and the two hits that reduced TcTDH activity to less than 20% (in *red*) were selected for further characterization. *B*, Inhibition of TcTDH by different concentrations of TCMDC-143160 (*purple*) and TCMDC-143463 (*green*). *C*, structure of TcTDH inhibitors, IC50 (and 95% confidence interval) values for TcTDH inhibition and EC50 values for *T*. *cruzi* amastigotes growth inhibition as previously reported (17). *D*, inhibition of *T*. *cruzi* epimastigotes viability by different concentrations of TCMDC-143160 (EC50 = 31 μM, 95%CI: 29–34 μM). *E*, concentration of acetate and glycine, end-products of L-threonine catabolism, excreted by starved *T*. *cruzi* epimastigotes feed exclusively with 5 mM L-threonine in the presence of 20 μM of TCMDC-143160 or 0,5% DMSO as a control (n = 3, paired *t* test, significant difference for *p* < 0.05).

To find an alternative chemical probe that could be used to assess the threonine degradation in *T*. *cruzi*, we decided to screen the 220 anti-*T*.*cruzi* phenotypic hits provided by GSK in the Chagas Box collection. The screening assay coupled the recombinant TcTDH and diaphorase reactions. The NADH produced during the oxidation of threonine to 2-keto-butyrate was used by diaphorase to catalyze the reduction of resazurin to resorufin, the fluorescent probe. It should be noted that Chagas Box samples were tested for diaphorase inhibition and no inhibitors were found (data not shown). The initial screen identified TCMDC-143160 and TCMDC-143463 as promising inhibitors for TcTDH (Fig. 4*A*), and the concentration-response assay returned low micromolar IC50 values for both compounds (Fig. 4*B*). Subsequently, a comparison between IC50 and EC50 values reported in Chagas Box (Fig. 4*C*) (17) suggested potential off-target activity for TCMDC-143463 and discourages its use as a probe for threonine catabolism in *T*. *cruzi*. The studies proceeded with TCMDC-143160, which exhibited compatible IC50 and EC50 values.

To assess the mode of action of TCMDC-143160 within the parasite, we first confirmed its activity on the growth of *T*. *cruzi* epimastigotes cultivated in LIT medium for 3 days. The compound demonstrated a clear dose-response profile, with an EC50 of 31 μM (Fig. 4*D*). Subsequently, we treated starved epimastigotes with a mild concentration of TCMDC-143160 (20 μM) and provided threonine (5 mM) as the only carbon source. After 24 h of treatment, the end-products of threonine catabolism were quantified by ^1^H-NMR spectroscopy. Parasites treated with TCMDC-143160 produced significantly less acetate and glycine than parasites in the control group (Fig. 4*E*), confirming the inhibition of TcTDH inside the parasite.

### TcTDH is activated by K^+^ and non-competitively inhibited by TCMDC-143160

**Figure 5:**
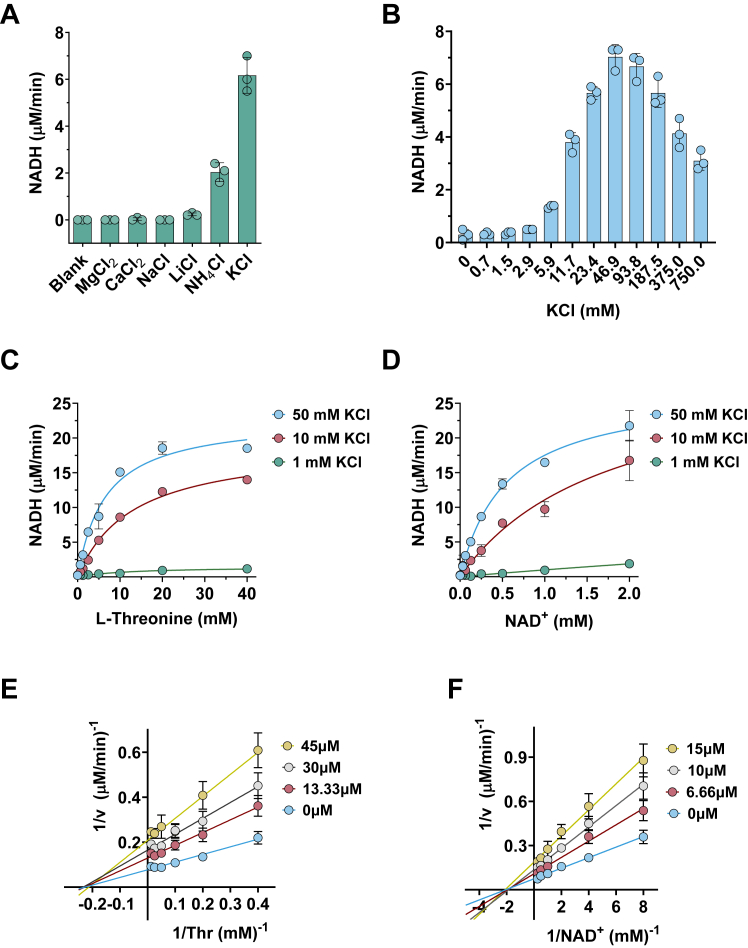
**TcTDH activation by potassium and inhibition by TCMDC143160**. *A*, effect of different salts (50 mM) on TcTDH activity. *B*, effect of different concentrations of KCl on TcTDH activity. (*C*) and (*D*) TcTDH Michaelis-Menten plots for L-threonine and NAD^+^ at different concentrations of KCl. (*E*) and (*F*) Double-reciprocal plots of TcTDH inhibition at different concentrations of TCMDC-143160 measured for both L-threonine and NAD^+^.

During the purification of the recombinant TcTDH, we noted that the presence KCl in the purification buffer improved protein stability and its specific activity. In the literature, Margaret Green was the first to characterize the activation of *S*. *aureus* TDH by potassium ions, back in the 1960s (16). These findings motivated us to further investigate the putative activation of TcTDH by metallic ions. Indeed, our results confirmed that TcTDH is activated by K^+^ and to a lesser extent by NH_4_^+^. Other monovalent and divalent cations, such as Li^+^, Na^+^, Ca^++^, and Mg^++^, exhibited no effect on the TcTDH activity (Fig. 5*A*). TcTDH activity was negligible in the absence of KCl. A significant increase in TcTDH activity was observed only at KCl concentrations higher than 5.9 mM, with maximal activity achieved at 46.9 mM KCl (Fig. 5*B*). To further characterize the activation of TcTDH by potassium, the enzyme kinetics parameters were measured at three different KCl concentrations, 1, 10, and 50 mM KCl those concentrations were selected to provide a low, an intermediary and the highest TcTDH activity (Fig. 5*C*). It is worth noting that due to the low TcTDH activity at 1 mM, kinetic parameters under this condition were poorly estimated, particularly when NAD^+^ was the variable substrate. Considering the kinetics parameters calculated for threonine as the variable substrate, *k*_cat_ and *k*_cat_/*K*_m_ increased 10-fold when KCl was elevated from 1 to 10 mM (Table 1) (Fig. 5, *C* and *D*). An examination of the kinetic parameters determined at 10 and 50 mM KCl suggests that the increase in catalytic efficiency is likely attributable to an increased affinity for substrates, as indicated by reduction in *K*_m_ values, without significant changes of *k*_cat_. Collectively, these results suggest that potassium ion acts as an allosteric activator of TcTDH.

**Table 1:** Kinect parameters for TcTDH at different concentrations of KCl (95% Confidence Interval indicated in parentheses)

KCl (mM)	*K*_m__Thr (mM)	k_cat__Thr (s^-1^)	k_cat_/*K*_m__Thr (μM^-1^.s^-1^)	*K*_m__NAD^+^ (mM)	k_cat__NAD^+^ (s^-1^)	k_cat_*/K*_m__NAD^+^ (μM^-1^.s^-1^)
1	12.6 (4.2–43)	0.5 (0.3–0.9)	0.04 (0.015–0.063)	16.0 (0–85.3)	5.6 (0–27.4)	0.3 (0.2–0.5)
10	13.1 (11.2–15.3)	6.4 (6.0–6.9)	0.49 (0.44–0.54)	1.8 (0.7–2.8)	10.3 (6.8–13.8)	5.8 (4.2–7.3)
50	6.6 (5.2–8.2)	7.7 (7.1–8.3)	1.2 (0.9–1.3)	0.5 (0.4–0.6)	9.0 (8.3–9.7)	16.6 (14.6–18.7)

Investigation of the mechanism of inhibition of TCMDC-143160 indicated it acts as a non-competitive inhibitor for both substrates (Fig. 5, *E* and *F*). Two independent *K*_i_ values were determined: one varying threonine at saturated concentration of NAD^+^ (*K*_i_-Thr = 26 μM) and another varying NAD^+^ at saturated threonine (*K*_i_-NAD^+^ = 11.2 μM). To elucidate the precise inhibition site, repeated attempts to soak and co-crystallize TCMDC-143160 in complex with TcTDH were performed. Several putative complex crystals were diffracted and their structures partially refined. Unfortunately, we have not found any evidence of electron density for the TCMDC-143160 in those structures. However, the investigation of TcTDH crystal structures revealed a putative binding site for potassium ion.

### The crystal structure of TcTDH and the importance of K^+^ coordination to the enzyme activity

Crystallographic structures of recombinant TcTDH without natural ligands (apo-TcTDH) and in complex with NAD^+^ and acetate (holo-TcTDH) were determined and refined to 2.1 and 1.73 Å resolution, respectively (Table S1). In both crystals, asymmetric units (ASU) comprise two polypeptide chains, related by a non-crystallographic symmetry two-fold axis. The two protomers in ASU correspond to the homodimeric biological unit. Size exclusion chromatography corroborated the homodimeric organization of TcTDH as the predominant quaternary structure in solution (data not shown). The quaternary structure and biological unit of TcTDH are analogous to those previously reported for other SDR TDHs (8, 9, 10, 11, 12). Due to insufficient electron density, certain residues were not incorporated in the final models. For apo-TcTDH, the initial 12 residues of chains A and B and the terminal two residues from the C-terminus of chain B were omitted. For the holo-TcTDH, the initial 12 residues from chain A and the initial 10 residues from chain B were omitted. In chain B, residues 57 to 60 and the terminal residue from the C-terminus were excluded from the final model.

**Figure 6:**
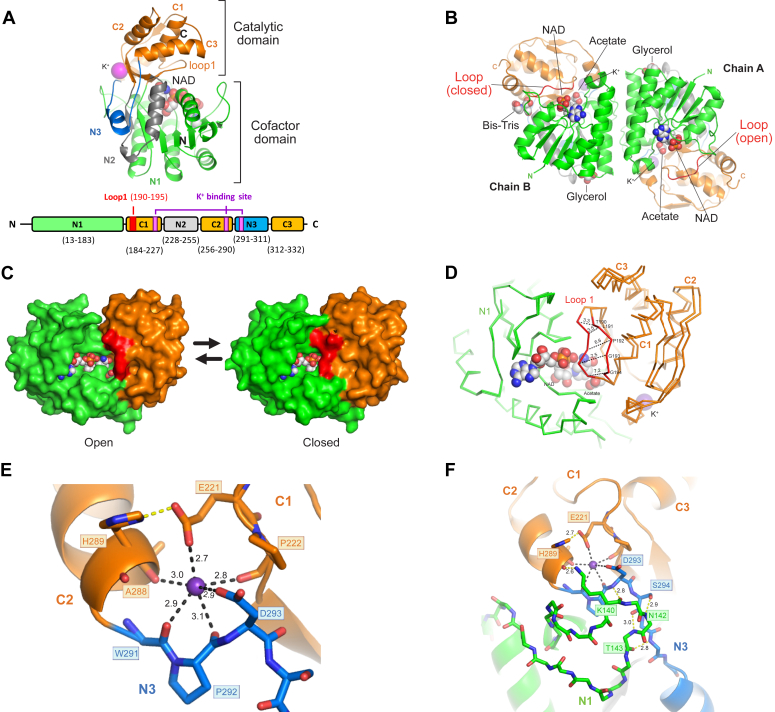
**Crystal structure of *T*. *cruzi* L-threonine dehydrogenase.***A*, monomer organization of TcTDH (pdbcode, 8gjb). Cofactor domain is formed by segments N1, N2 and N3, while the catalytic domain comprises segments C1, C2 and C3. *B*, TcTDH quaternary structure highlighting the different orientations of loop1, which control the access of NAD^+^ and acetate (in balls) to the catalytic site. *C*, surface representation of chains A and B with ligands (in balls) and loop-1 in *red*. *D*, superposition of chain *A* and *B*. Protomers were superimposed by the N-terminal domains. The largest Cα – Cα distances are observed for loop-1 residues. *E*, Potassium coordination and (*F*) remote switch region around the potassium binding site. Figures were prepared in PYMOL v.2.5 (Schrodinger, LLC, New York, NY, USA).

The tertiary structure of TcTDH comprises two domains composed of alternating protein segments. The cofactor-binding domain initiates in the N-terminal and is formed by three segments, N1 (13–183), N2 (228–225), and N3 (291–311). Concurrently, the catalytic domain is formed by segments C1 (184–227), C2 (256–290) and C3 (312–332) (Fig. 6*A*). Segments N2 and N3, from the cofactor binding domain, are encoded between segments of the catalytic domain, resulting in an alternating segmentation pattern. The cofactor-binding domain adopts a characteristic Rossman motif and contains both catalytic residues, Thr-130 and Tyr-155. These residues are highly conserved in other SDR TDHs (Fig. S3) and were proposed to function in the hydrogen abstraction of the threonine side chain β-hydroxyl (9). The homodimeric organization of TcTDH is facilitated by non-covalent interactions between residues in N1 segments of adjacent cofactor-binding domains (Fig. 6*B*). The catalytic domain exhibits a α+β fold and regulates access to the catalytic site through displacement of Loop-1 (residues 190–194), which is observed in open and closed conformations in chains A and B of the holo-TcTDH structure, respectively (Fig. 6*C*). Superposition of alpha carbons from chains A and B of holo-TcTDH yields an overall rmsd of 0.92 Å with greater displacements localized at Loop-1 (Fig. 6*D*).

The potassium-binding site is situated opposite to the catalytic site. According to Gohara D. W. and Di Cera E. (18), potassium ion coordination by proteins exhibits an octahedral organization with six or seven oxygen atoms at bond distance between 2.8 ± 0.2 Å or 2.9 ± 0.3 Å, respectively. In TcTDH, K^+^ is observed in octahedral coordination established through interactions with residues in the catalytic domain (Glu-221, Pro-222, and Ala-288) and cofactor-binding domain (Trp-291, Pro-292, and Asp-293) (Fig. 6*E*). Four interactions are established between K^+^ and the main chain carbonyl of Pro-222, Ala-288, Trp-291, and Pro-292, and two interactions with the side chain carboxyl of Glu-221 and Asp-293. Although not directly interacting with K^+^, residues His-289, Lys-140, Asn-142, and Thr-143 formed a secondary layer of hydrogen bonds that imposed additional constraints on this region (Fig. 6*F*). The nitrogen side chain and main chain carbonyl of His-289 interact with the side chain carboxyl of Glu-221 and with the side chain amine from Lys-140, respectively. Besides, the main chain carboxyl of Lys-140 is involved in a hydrogen bond with the main chain amide of Asp-293. The main chain amides of Asn-142 and Thr-143 form hydrogen bonds with the hydroxyl side chain and main chain carbonyl of Ser-294, respectively. Thr-143 forms a second hydrogen bond *via* its carbonyl with the amide of Asn-296 in the protein backbone. Collectively, these interactions connect the largest loop of the N1 segment with residues in the N3 segment and with the potassium-binding site.

To confirm the location of the K^+^ binding site and its relevance for TcTDH activity, five mutants were engineered by site-directed mutagenesis. TcTDH-E221 A and D293 A mutants were designed to evaluate local constraints imposed by potassium coordination. The other mutants, H289 A, K140 A, and S294 A, are not directly involved in K^+^ coordination, but they participate in a secondary layer of interactions around the metal binding site. As anticipated, the mutant TcTDH-E221 resulted in the most significant reduction of TcTDH-specific activity, exceeding 1000-fold when compared to the wild-type enzyme (Table 2). Contrary to expectations, TcTDH-D293 exhibited the least impact, decreasing enzyme activity by approximately 10-fold. Given its solvent accessibility and absence of other relevant interactions, we speculate that a water molecule could potentially fulfill the potassium coordination in this mutant. The other mutants reduced TcTDH activity by more than 100-fold, confirming the significance of loop 140 to 143 in the allosteric regulation of TcTDH.

**Table 2:** Specific activity of TcTDH mutants measured as produced NADH in the presence of 10 mM L-threonine, 0.5 mM NAD^+^ and 50 mM KCl. 95% Confidence Interval indicated in parentheses

TcTDH mutants	U (nmols/min/mg)	Normalized activity (%)
E221 A	0.9 (0.7–1.1)	0,02
K140 A	15 (9.3–20)	0,3
H289 A	23 (22–24)	0,5
S294 A	16 (12–21)	0,3
D293 A	578 (551–605)	12,3
wild-type	4697 (4535–4859)	100

## Discussion

**Figure 7:**
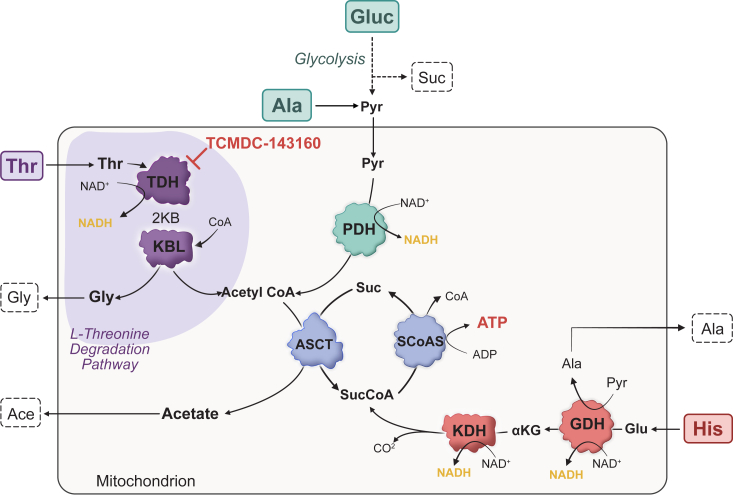
**Mitochondrial pathways associated to threonine, alanine, histidine and glucose catabolism by *T*. *cruzi* epimastigotes under nutrient restrict condition.** The filled and dashed boxes indicate consumed and excreted metabolites, respectively. L-threonine dehydrogenase (TDH), 2-amino-3-ketobutyrate CoA ligase (KBL), pyruvate dehydrogenase (PDH), acetate:succinate CoA-transferase (ASCT), succinyl-CoA synthetase (SCoAS), α-ketoglutarate dehydrogenase, (KDH) and glutamate dehydrogenase (GDH). (created with Affinity Designer (Version 1.10.6.1665), Serif Ltd).

Threonine is a valuable nutrient for trypanosomes, especially under low-carbohydrate conditions. In *T*. *brucei* procyclic forms, the threonine degradation pathway was demonstrated to occur in the parasite mitochondrion, where it produces glycine and acetyl-CoA in equal amounts (3). Acetyl-CoA is then converted into acetate by ACH or the ACST/SCoAS catalytic cycle, but only the latter cycle produces ATP by substrate-level phosphorylation (4). SCoAS knockout in *T*. *brucei* bloodstream forms increases parasite dependency of the ADP/ATP carrier, which transfers ATP from cytosol into the mitochondrion to be hydrolyzed by the FoF1-ATP synthase in a reverse reaction, pumping out H^+^ to maintain the organelle membrane potential and parasite viability (19). It is worth noting that the ACST/SCoAS cycle requires acetyl-CoA and succinyl-CoA to spin out. Acetyl-CoA can be generated from pyruvate and threonine, whereas succinyl-CoA is produced by the catabolism of specific amino acids through the activity of the α-ketoglutarate dehydrogenase complex (Fig. 7). Our results indicate that histidine, glutamine, asparagine, aspartate, and proline, which are consumed by *T*. *cruzi* from the LIT medium, could provide succinyl-CoA to the ACST/SCoAS cycle. During the exponential growth phase, the catabolism of histidine, glutamine, and asparagine may produce succinyl-CoA, while the catabolism of threonine and carbohydrates generates acetyl-CoA. Under such nutrient-rich conditions, acetate excretion reaches its highest rate, and excess carbohydrates are catabolized to succinate in glycosomes and then excreted to the extracellular medium. Alanine excretion is also high and may be derived from pyruvate through the activity of alanine or tyrosine aminotransferases (20, 21) and glycosomal alanine dehydrogenase (22). By the fifth day, when carbohydrates and threonine are depleted from the medium, a shift in metabolism is observed and alanine, aspartate, and proline begin to be consumed. Under nutrient-restricted conditions, alanine may sustain acetyl-CoA production, while aspartate and proline provide succinyl-CoA. The catabolism of alanine by starved parasites was previously investigated by Girard *et al*. (23), who observed significant ATP production, while only 10% of the consumed amino acid was oxidized to CO_2_. In our experiments, starved parasites rescued with alanine showed increased acetate excretion. Together, these observations suggest that under nutrient restriction, the catabolism of alanine generates ATP in the ACST/ScoAS cycle, which is used to maintain the mitochondrion membrane potential and consequently, the parasite viability.

Our results also confirm the function of the threonine degradation pathway and ACST/ScoAS cycle in *T*. *cruzi* epimastigotes rescued with threonine from a 24h starvation condition. In contrast to *T*. *brucei* (3), we observed a higher excretion of glycine than acetate, and we attribute this unbalanced glycine/acetate excretion to a lower intracellular succinyl-CoA concentration, as previously observed by Barisón JM and collaborators, for *T*. *cruzi* epimastigotes in the stationary phase (24). Without sufficient succinyl-CoA, the ACST/ScoAS cycle runs slowly, limiting the production of acetate and accumulating glycine and acetyl-CoA, the upstream metabolites in the threonine degradation pathway.

To further investigate the relevance of threonine catabolism in *T*. *cruzi*, we used TCMDC-143160, which inhibits the recombinant TcTDH and the growth of both epimastigote (present work) and amastigote forms of the parasite (17). We validated the intracellular mode of action of TCMDC-143160 by demonstrating a significant reduction in acetate and glycine excretion in *T*. *cruzi* epimastigotes. Our findings suggest that threonine is an important nutrient for parasite growth, particularly under limited nutrient conditions. It is noteworthy that once inside its mammalian host cell, the parasite competes with numerous native mitochondria for available nutrients. Given that threonine is not degraded by human mitochondria due to the absence of TDH, it is reasonable to speculate that it may be a valuable energy source for the parasite.

Another interesting aspect of TDH activity is its allosteric regulation. The potassium-binding site of TcTDH superposes with a methylation site in mTDH (11), both located between the catalytic and cofactor-binding domains. Structural superposition showed that Lys-140 in TcTDH, which participates in K^+^ coordination, coincides with Lys-180 in mTDH, whose methylation increases enzyme activity. This suggests that regardless of the mechanism, either by metal-binding or methylation, modification in this protein region controls enzyme activity and, consequently, the overall threonine catabolism. In conclusion, we recommend the use of TCMDC-143160 as a probe to investigate threonine catabolism in trypanosomes. However, as we do not know yet the precise binding site of TCMDC-143160, it is important to confirm its inhibition of the target enzyme before advancing to cell-based experiments. Indeed, our findings point to TcTDH as the first target described for TCMDC-143160, and so far, we cannot exclude the existence of secondary off-targets.

## Experimental procedures

### *T*. *cruzi* growth

*T*. *cruzi* (Dm28c, DTU-TcI) epimastigotes were maintained in LIT medium supplemented with 10% fetal bovine serum (FBS) (Vitrocell, Campinas, SP, Brazil), 1% Penicillin-Streptomycin solution 10,000 U/ml (Gibco) and bovine hemin (Sigma-Aldrich), at 28°C. A growth curve was initiated using *T*. *cruzi* epimastigotes at density of 1 × 10^7^ parasites per milliliter of LIT medium. Three samples of 1 mL each were collected every 2 days from the same culture flask, which was incubated for 13 days. After parasite counting in Neubauer chamber, samples were centrifuged at 13,000 rpm for 10 min and the supernatant was stored at −20°C, for ^1^H-NMR exometabolomic profiling. Samples of non-inoculated LIT medium were used to represent time zero.

### Starvation recovery assay

*T*. *cruzi* epimastigotes in early exponential growth were harvested by centrifugation, washed twice with PBS (Gibco), and resuspended in the same buffer to a density of 0.5 x 10^8^ parasites per mL. After a starvation period of 24 h, 1 mL samples were transferred to a 24-well plate and supplemented with L-threonine, L-alanine, L-histidine, or D-glucose (5 mM). A negative control was maintained in PBS, without supplementation, while a positive control was harvested by centrifugation and transferred to 1 ml of fresh LIT medium. After a recovery period of 24 h, samples (0.5 ml) were centrifuged at 13,000 rpm for 10 min and the supernatant was stored at −20°C, for ^1^H-NMR exometabolomic profiling. For the resazurin redox assay, samples (0.15 ml) were transferred to a 96-well plate, supplemented with 30 μl of resazurin at a concentration of 0.15 mg/ml, and incubated for 3h at 37 °C. Resazurin reduction was quantified by fluorescence readout (Ex = 570 nm; Em = 590 nm). Data were normalized using LIT medium in the absence and presence of parasites as 0 and 100%, respectively. A comparison of normalized means of distinct nutrients *versus* PBS treatment was conducted using ordinary one-way ANOVA followed by Dunnett's test for significance (*p* < 0.05 for a significant difference). To analyze the effect of TCMDC-143160 on threonine catabolism, starved parasites were treated with 20 μM (0.5% DMSO) of the compound for 2 h before rescue with L-threonine (5 mM). Acetate and glycine excreted to the extracellular medium were quantified by ^1^H-NMR. Differences from three independent experiments were compared by paired *t* test and *p*-value < 0.05 indicate significant difference.

### ^1^H-NMR exometabolomic

Samples from growth curve and starvation recovery assays were thawed in ice bath, centrifuged at 13,000 rpm for 10 min, and filtered in AMICON ULTRA-0.5 ml 3 kDa (MERCK, cat# UFC5003BK). 0.3 ml of ultrafiltered samples were mixed with 0.24 ml deuterium oxide (Sigma-Aldrichcat#151882) and 0.06 ml of 5 mM TMSP-d_4_ (MERCK, cat# 269913) and transferred to 5 mm glass tube for ^1^H-NMR data acquisition.

^1^H-NMR spectra were acquired using a Varian/Agilent Inova spectrometer (Agilent Technologies Inc) equipped with a triple-resonance cold probe and operating at a proton resonance frequency of 599.880 MHz. For each spectrum, 512 scans with 32,000 data points were collected over a width of 8000 Hz. A 1.5 s relaxation delay was incorporated between scans, during which a continual water pre-saturation radio frequency field was applied to eliminate residual signal. A 0.5 Hz line-broadening function was used to reduce signal-to-noise ratio and facilitate metabolite identification. The water signal was suppressed, and spectra calibrated using the signal of 0.5 mM TMSP-d_4_ (Merck, cat# 269913) as a reference. Samples metabolites were identified and quantified using NMR Suite software version 8.1 (Chenomx Inc). Metabolites concentration data were analyzed in MetaboAnalyst 5.0 (25) and GraphPad Prism 9.5.0 .

### TcTDH expression, purification, and site-directed mutagenesis

The sequence of the putative TcTDH from CL Brener Esmeraldo-like strain (tritrypDB: TcCLB.507923.10) was used to design a codon optimized gene for overexpression in *E*. *coli*. The optimized gene was flanked by BamHI and XhoI restriction sites for insertion in pET28a vector. Synthesis and subcloning services were purchased from FastBio (Ribeirão Preto, São Paulo, Brazil). The vector pET28a containing the optimized gene was used to transform *E*. *coli* BL21 (DE3) and grown in autoinducing medium ZYM-5052 (26), supplemented with 30 μg/ml kanamycin for 72 h at 20°C and 200 rpm. Cells were harvested and resuspended in 40 ml of buffer A (50 mM sodium phosphate pH 7.6, 100 mM NaCl, 50 mM KCl, 5% glycerol and 2 mM imidazole), supplemented with 1 mg/ml lysozyme. Cells were disrupted by sonication and total soluble protein fraction clarified by centrifugation. The enzyme was purified by nickel affinity chromatography using a HisTrap HP column (GE Healthcare Life Technologies, Pittsburgh, PA) equilibrated with buffer A. TcTDH was eluted from the column in buffer A supplemented with 200 mM imidazole and 2 mM dithiothreitol (DTT). Next, TcTDH was subjected to size exclusion chromatographic in a HiLoad Superdex 200 16/60 column (GE Healthcare Life Technologies) equilibrated with GF buffer (20 mM sodium phosphate pH 7.6, 40 mM NaCl and 40 mM KCl). The eluted fractions were analyzed by dynamic light scattering (DLS) and SDS-PAGE 15%. Fresh samples were used for crystallization trials or frozen in 10% glycerol for enzymatic assays. The mutants TcTDH-D293 A, TcTDH-E221 A, TcTDH-H289 A, TcTDH-K140 A, and TcTDH-S294 A were obtained by site-directed mutagenesis using the oligonucleotides indicated in Supplementary Information (Table S2). The mutated genes were subcloned into the vector pET28a-SUMO between BamHI and XhoI endonucleases restriction sites. Mutations were confirmed by gene sequencing by Sanger method. The expression and nickel affinity chromatography purification steps followed the same procedure described for the wild-type TcTDH. Two additional purification steps were introduced: incubation with ULP-1 protease (1U/ml) for 16 h at 4 °C and a second nickel affinity chromatography for elimination of the his-SUMO-tag. The mutants were frozen in buffer containing 10% glycerol for enzymatic assays.

### TcTDH enzyme kinetics

TcTDH activity was measured following the formation of NADH fluorescence intensity (Ex = 340 nm; Em = 485 nm) at 25°C, in black 384-wells microplates, at a final volume of 50 μl. The NADH fluorescence standard curve was used to convert relative fluorescence intensity measurements into product formation rates, expressed in NADH micromolar produced per minute (μM/min). TcTDH activation by metallic ions was evaluated by adding 50 mM of MgCl_2_, CaCl_2_, NaCl, LiCl, NH_4_Cl or KCl to the Reaction Buffer (50 mM KCl, 0.01% Triton X-100 and 50 mM sodium phosphate buffer, pH 7.6) containing 10 mM L-Threonine, 500 μM NAD^+^ and 50 nM TcTDH. Potassium activation was performed by varying KCl from 750 to 0.7 mM. Kinetics parameters were measured at 1, 10, and 50 mM of KCl. The $K_{m}^{app}$ for L-threonine was obtained by varying the substrate from 40 mM to 0.625 mM, in the presence of 1 mM of NAD^+^. $K_{m}^{app}$ for NAD^+^ was determined varying the cofactor concentration from 2 mM to 31.25 μM, in the presence of 20 mM of L-Threonine. Michaelis-Menten constants were calculated by nonlinear regression in GraphPad Prism 9.5.0. Potassium activation of TcTDH-D293 A, TcTDH-E221 A, TcTDH-K140 A, TcTDH-H289 A and TcTDH-S294 A mutants were evaluated in the same conditions as described for the TcTDH wild-type, but only at KCl concentration at 50 mM.

### TcTDH inhibition assay

To screen Chagas Box library for TcTDH inhibitors, compounds were assayed at a single concentration of 10 μM. Compounds were provided by GSK in 384-wells ready-to-use assay microplate containing 25 nl of each sample at 10 mM (100% DMSO). Next, 20 μl of Reaction buffer (as described above) supplemented with 12.5 mM L-Threonine, 625 μM NAD^+^, 25 μM resazurin, 1.2 U/ml diaphorase was dispensed into wells and the reaction initiated by addition of 5 μl of 0.25 μM TcTDH (in Reaction Buffer). After 15 min, resorufin fluorescence (Ex = 570 nm; Em = 590 nm) was measured in a plate reader (CLARIOstar, BMG LabTech). Data were normalized and plotted using GraphPad Prism 9.5.0.

To determine the concentration necessary to inhibit 50% of TcTDH activity (IC50), TCMDC-143160 and TCMDC-143463 stock solutions (5 mM) were serially diluted in DMSO, applying a ½-log dilution factor and 1 μl transferred to 384-wells assay microplate. Ten concentration points were plated for each compound. Next, 45 μl of Reaction buffer (as described above) supplemented with 10 mM L-threonine, 500 μM NAD^+^, 20 μM resazurin, 1 U/ml diaphorase solution was dispensed into wells and the reaction initiated by addition of 5 μl of 0.5 μM TcTDH (in Reaction Buffer). Resorufin fluorescence was recorded every 20 s for 5 min and maximum reaction velocities were calculated in the plate reader (CLARIOstar, BMG LabTech). TcTDH activity was normalized, and the IC50 was calculated using GraphPad Prism 9.5.0.

### TCMDC-143160 inhibition mechanism

TDH assays were performed in black 96 well plates, using 100 μl of Reaction Buffer containing 50 nM of TcTDH. TCMDC-143160 inhibition mechanism and *K*_i_ values were determined by measuring reaction rates varying L-threonine from 80 to 0.625 mM or NAD^+^ from four to 0.0313 mM, while keeping the other substrate at saturating concentrations, 5 mM of NAD^+^ or 80 mM of L-threonine, respectively. Max slopes of time-course curves obtained by measuring NADH fluorescence intensity (Ex = 340 nm; Em = 485 nm) were used to plot double-reciprocal Lineweaver-Burk representation. Kinetic constants were calculated by nonlinear regression analysis in GraphPad Prism 9.5.0 (San Diego, CA, USA).

### *T*. *cruzi* epimastigote inhibition assay

1 × 10^6^ cells of *T*. *cruzi* epimastigotes were incubated in 147 μl LIT medium in 96 well plate, in the presence of compounds TCMDC-143160, Qc1 and Benznidazole, ranging from 80 to 0.62 μM (2% DMSO), for 72 h at 28°C. Following the incubation period, 30 μl of resazurin solution (0.15 mg/ml) was added to each well and incubated for 3 h before fluorescence readout (Ex = 570 nm; Em = 590 nm). Data were normalized using 80 μM Benznidazole and non-treated wells as 0 and 100% of viability. The resulting data were fitted to a sigmoidal curve and analyzed in GraphPad Prism 9.5.0. This experiment was done in biological triplicate.

### *T*. *cruzi* intracellular image-based assay

Methodology for intracellular image-based assay has been previously described (27). Briefly, *T*. *cruzi* trypomastigotes collected from LLC-MK2 (ATCC code: CRL-1446) supernatant were used to infect rat cardiomyocytes H9c2 (BCRJ code: 0146), overnight, washed and incubated for addition 48 h in fresh DMEM medium. Cells at density of 1.5 x 10^3^ were transferred to 384 wells-microplates, containing 0.2 μl of acoustically transferred samples (assay volume of 50 μl and 0.4% DMSO). After 72 h incubation, cells were washed, fixed with 4% PFA and stained with Hoechst 33342 (4 μg/ml in PBS). Five images per well were acquired with the fluorescence microscope Operetta (PerkinElmer, Hamburg, DE) using a 20x long WD objective and processed in Columbus software (PerkinElmer, Hamburg, DE). Total cells and infection ratio data were analyzed and plotted in GraphPad Prism 9.5.0.

### TDH crystal structure

Apo-TcTDH and holo-TcTDH crystals were obtained by the sitting-drop vapor diffusion method at 18°C. For holo-TcTDH complex, 10 mM NAD^+^ was added to the freshly purified TcTDH at 10 mg/ml. Crystals were obtained by mixing 1 μl of protein solution with an equal volume of reservoir solution, containing: 25% PEG 3350, 200 mM ammonium acetate and 100 mM Bis-Tris pH 5.3 to 5.5. Crystals were transferred to a cryoprotectant solution, made of reservoir solution plus 25% glycerol and then frozen in liquid nitrogen. X-ray diffraction experiments were performed at MANACÁ beamline (SIRIUS-CNPEM, Campinas, Brazil), equipped with a PILATTUS 2M X-ray detector and wavelength set to 0.977 Å. Diffraction datasets were obtained at 100 K, using fine slicing of 0.1° per image, exposure time of 0.07 s and measuring a total of 360°. X-ray diffraction data were automatically processed with XDS (28), AIMLESS (29) using CCP4i2 suite v.7.0.078 (30). The structure was solved by molecular replacement, using the TbTDH (PDB 5L9A) as search model and PHASER software (31). The restrained refinement was made with REFMAC (32) and manual rebuilding was done in COOT (33). Crystallographic statistics and PDB codes are reported in Table S1. Molecular figures were prepared in PYMOL v.2.5 (Schrodinger, LLC, New York, NY, USA).

## Data availability

The coordinates and structure factors for the apo-TcTDH (PDB code: 8GIL) and holo-TcTDH (PDB code: 8GJB) structures have been deposited in the PDB.

## Supporting information

This article contains supporting information (34).

## Conflict of interest

The authors declare that they have no conflicts of interest with the contents of this article.

## References

[1] World Health Organization (WHO) (2023).

[2] Souza R.O.O., Damasceno F.S., Marsiccobetre S., Biran M., Murata G., Curi R. (2021). Fatty acid oxidation participates in resistance to nutrient-depleted environments in the insect stages of Trypanosoma cruzi. Plos Pathog..

[3] Millerioux Y., Ebikeme C., Biran M., Morand P., Bouyssou G., Vincent I.M. (2013). The threonine degradation pathway of the Trypanosoma brucei procyclic form: the main carbon source for lipid biosynthesis is under metabolic control. Mol. Microbiol..

[4] Millerioux Y., Morand P., Biran M., Mazet M., Moreau P., Wargnies M. (2012). ATP synthesis-coupled and -uncoupled acetate production from acetyl-CoA by mitochondrial acetate:Succinate CoA-transferase and acetyl-CoA thioesterase in Trypanosoma. J. Biol. Chem..

[5] Tielens A.G.M., van Hellemond J.J. (2009). Surprising variety in energy metabolism within Trypanosomatidae. Trends Parasitol..

[6] Ishikawa K., Higashi N., Nakamura T., Matsuura T., Nakagawa A. (2007). The first crystal structure of l-threonine dehydrogenase. J. Mol. Biol..

[7] Bowyer A., Mikolajek H., Stuart J.W., Wood S.P., Jamil F., Rashid N. (2009). Structure and function of the l-threonine dehydrogenase (TkTDH) from the hyperthermophilic archaeon Thermococcus kodakaraensis. J. Struct. Biol..

[8] Yoneda K., Sakuraba H., Muraoka I., Oikawa T., Ohshima T. (2010). Crystal structure of UDP-galactose 4-epimerase-like l-threonine dehydrogenase belonging to the intermediate short-chain dehydrogenase-reductase superfamily. FEBS. J..

[9] Yoneda K., Sakuraba H., Araki T., Ohshima T. (2012). Crystal structure of binary and ternary complexes of archaeal UDP-galactose 4-epimerase-like L-threonine dehydrogenase from Thermoplasma volcanium. J. Biol. Chem..

[10] Nakano S., Okazaki S., Tokiwa H., Asano Y. (2014). Binding of NAD+and L-threonine induces stepwise structural and flexibility changes in cupriavidus necator L-threonine dehydrogenase. J. Biol. Chem..

[11] He C., Huang X., Liu Y., Li F., Yang Y., Tao H. (2015). Structural insights on mouse l-threonine dehydrogenase: a regulatory role of Arg180 in catalysis. J. Struct. Biol..

[12] Adjogatse E., Erskine P., Wells S.A., Kelly J.M., Wilden J.D., Chan A.W.E. (2018). Structure and function of L-threonine-3-dehydrogenase from the parasitic protozoan Trypanosoma brucei revealed by X-ray crystallography and geometric simulations. Acta Crystallogr. D Struct. Biol..

[13] Han C., Gu H., Wang J., Lu W., Mei Y., Wu M. (2013). Regulation of L-threonine dehydrogenase in somatic cell reprogramming. Stem Cells.

[14] Wang J., Alexander P., Wu L., Hammer R., Cleaver O., McKnight S.L. (1979). Dependence of mouse embryonic stem cells on threonine catabolism. Science.

[15] Alexander P.B., Wang J., McKnight S.L. (2011).

[16] Green M.L. (1964). The activation of l-threonine dehydrogenase by potassium ions. Biochem. J..

[17] Peña I., Pilar Manzano M., Cantizani J., Kessler A., Alonso-Padilla J., Bardera A.I. (2015). New compound sets identified from high throughput phenotypic screening against three kinetoplastid parasites: an open resource. Scientific Rep..

[18] Gohara D.W., Di Cera E. (2016). Molecular mechanisms of enzyme activation by monovalent cations. J. Biol. Chem..

[19] Taleva G., Husová M., Panicucci B., Hierro-Yap C., Pineda E., Biran M. (2023). Mitochondrion of the Trypanosoma brucei long slender bloodstream form is capable of ATP production by substrate-level phosphorylation. Plos Pathog..

[20] Duschak V.G., Cazzulo J.J. (1991). Subcellular localization of glutamate dehydrogenases and alanine aminotransferase in epimastigotes of Trypanosoma cruzi. FEMS Microbiol. Lett..

[21] Nowicki C., Montemartini M., Duschak V., SantomÃ© J., Cazzulo J.J. (1992). Presence and subcellular localization of tyrosine aminotransferase and p-hydroxyphenyllactate dehydrogenase in epimastigotes of Trypanosoma cruzi. FEMS Microbiol. Lett..

[22] Acosta H., Dubourdieu M., Quiñones W., Cáceres A., Bringaud F., Concepción J.L. (2004). Pyruvate phosphate dikinase and pyrophosphate metabolism in the glycosome of Trypanosoma cruzi epimastigotes. Comp. Biochem. Physiol. B Biochem. Mol. Biol..

[23] Girard R.M.B.M., Crispim M., Alencar M.B., Silber A.M. (2018). Uptake of l-alanine and its distinct roles in the bioenergetics of trypanosoma cruzi. mSphere.

[24] Barisón M.J., Rapado L.N., Merino E.F., Pral E.M.F., Mantilla B.S., Marchese L. (2017). Metabolomic profiling reveals a finely tuned, starvationinduced metabolic switch in Trypanosoma cruzi epimastigotes. J. Biol. Chem..

[25] Pang Z., Chong J., Zhou G., De Lima Morais D.A., Chang L., Barrette M. (2021). MetaboAnalyst 5.0: narrowing the gap between raw spectra and functional insights. Nucleic Acids Res..

[26] Studier F.W. (2005). Protein production by auto-induction in high-density shaking cultures. Protein Expr. Purif..

[27] Fredo Naciuk F., Do Nascimento Faria J., Goncąlves Eufrásio A., Torres Cordeiro A., Bruder M. (2020). Development of selective steroid inhibitors for the glucose-6-phosphate dehydrogenase from trypanosoma cruzi. ACS Med. Chem. Lett..

[28] Kabsch W. (2010). Xds. Acta Crystallogr. D Biol. Crystallogr..

[29] Evans P. (2006). Scaling and assessment of data quality. Acta Crystallogr. D Biol. Crystallogr..

[30] Winn M.D., Ballard C.C., Cowtan K.D., Dodson E.J., Emsley P., Evans P.R. (2011). Overview of the CCP4 suite and current developments. Acta Crystallogr. D Biol. Crystallogr..

[31] McCoy A.J., Grosse-Kunstleve R.W., Adams P.D., Winn M.D., Storoni L.C., Read R.J. (2007). Phaser crystallographic software. J. Appl. Crystallogr..

[32] Kovalevskiy O., Nicholls R.A., Long F., Carlon A., Murshudov G.N. (2018). Overview of refinement procedures within REFMAC 5: utilizing data from different sources. Acta Crystallogr. D Struct. Biol..

[33] Emsley P., Lohkamp B., Scott W.G., Cowtan K. (2010). Features and development of coot. Acta Crystallogr. D Biol. Crystallogr..

[34] Cosentino RO, Agüero F (2012). A simple strain typing assay for Trypanosoma cruzi: discrimination of major evolutionary lineages from a single amplification product. PLoS Negl Trop Dis.

