# Supplementary material for: Inhibition of L-threonine dehydrogenase from Trypanosoma cruzi reduces glycine and acetate production and interferes with parasite growth and viability
Source: J Biol Chem. 2024 Dec 13;301(2):108080. doi: 10.1016/j.jbc.2024.108080 (PMC11910319; doi:10.1016/j.jbc.2024.108080)
Supplement: Supporting Information [file mmc1.docx]

Inhibition of L-threonine dehydrogenase from *Trypanosoma cruzi* reduces glycine and acetate production and interferes with parasite growth and viability.

Jessica do N. Faria^1,*^, Amanda G. Eufrásio^1,2,*^, Michelle Fagundes^1^, Angel Lobo-Rojas^1^, Letícia Marchese^1^, Caio Cesar de Lima Silva^1^ Eduardo H. S. Bezerra^1^, Gustavo F. Mercaldi^1^, Marcos R. Alborghetti^1^, Mauricio L. Sforca^1^ and Artur T. Cordeiro^1^

**Supporting information**

**Material included**

***Trypanosoma cruzi* strain identification**

**Fig**. S1 Heatmap plot for time-course metabolites profiles.

**Fig.** S2 Metabolites concentration plots.

**Fig.** S3 Alignment of TDH homologous sequences.

**Fig.** S4 Alignment of TcSC5D sequenced from the strain used in this study (TcSC5D_Seq) and strain Dm28c (gene BCY84_02439)

**Table S1.** TcTDH crystallography statistics.

**Table S2.** Oligonucleotides used for site-directed mutagenesis of TcTDH**.**

**Fig.** S1 Heatmap depicting Pearson's correlation hierarchical clustering analysis, utilizing Ward's linkage, for 54 metabolites identified by proton-NMR in LIT medium employed for the cultivation of *T. cruzi* epimastigotes over a thirteen-day period. The exponential and stationary growth phases are denoted in green and red, respectively. Identified metabolites are presented in rows, with auto-scaled concentrations color-coded according to z-score values (scale provided in the top right). Three technical replicates were analyzed for the same biological sample at distinct time points.


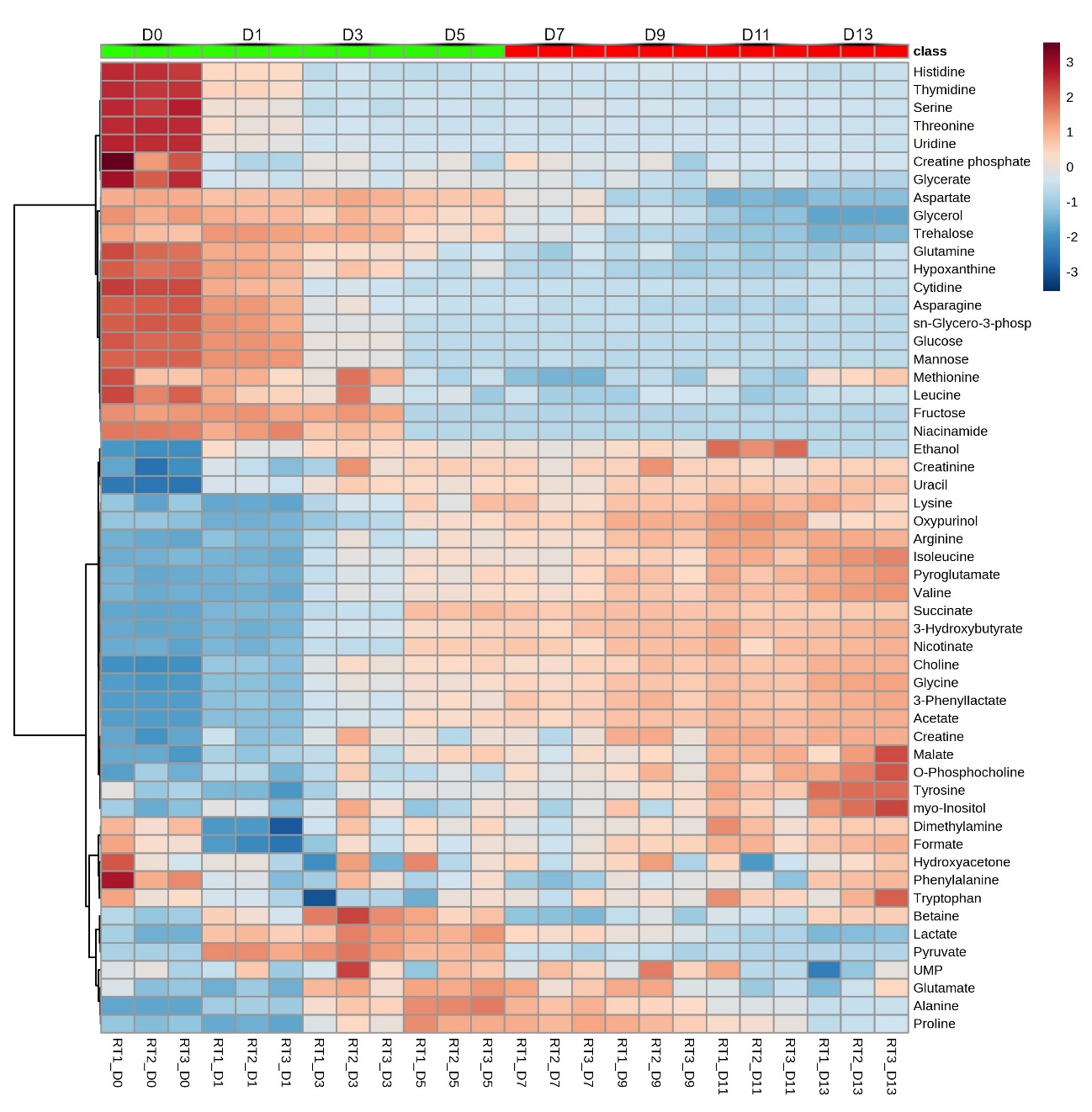


**Fig.** S2 Individual plots depicting metabolite concentrations detected in LIT medium used for the cultivation of *T. cruzi* epimastigotes over a thirteen-day period. Each data point represents the mean value derived from three technical replicate samples, all prepared from a single cultured flask at different time points.


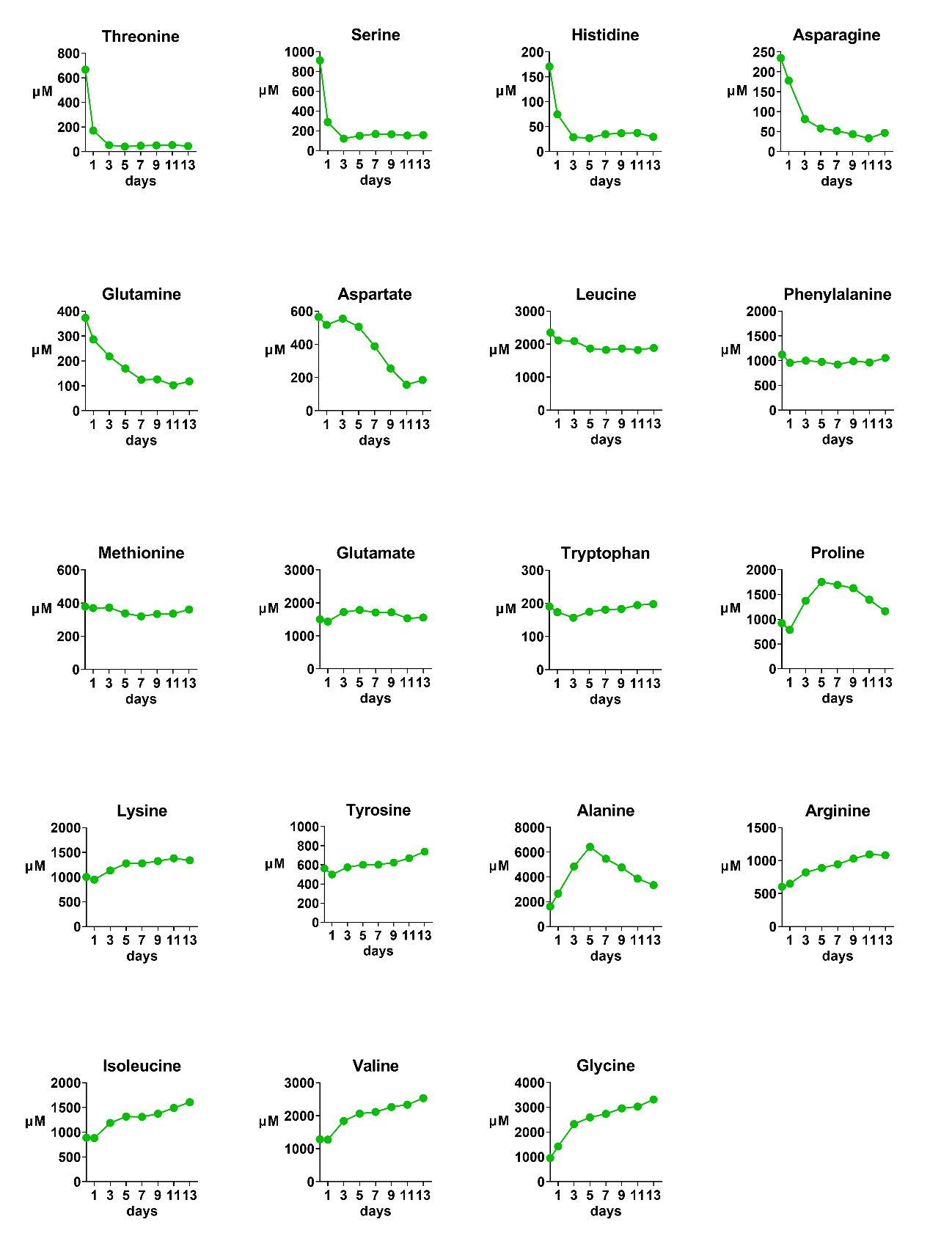


Continuation of Fig. S2


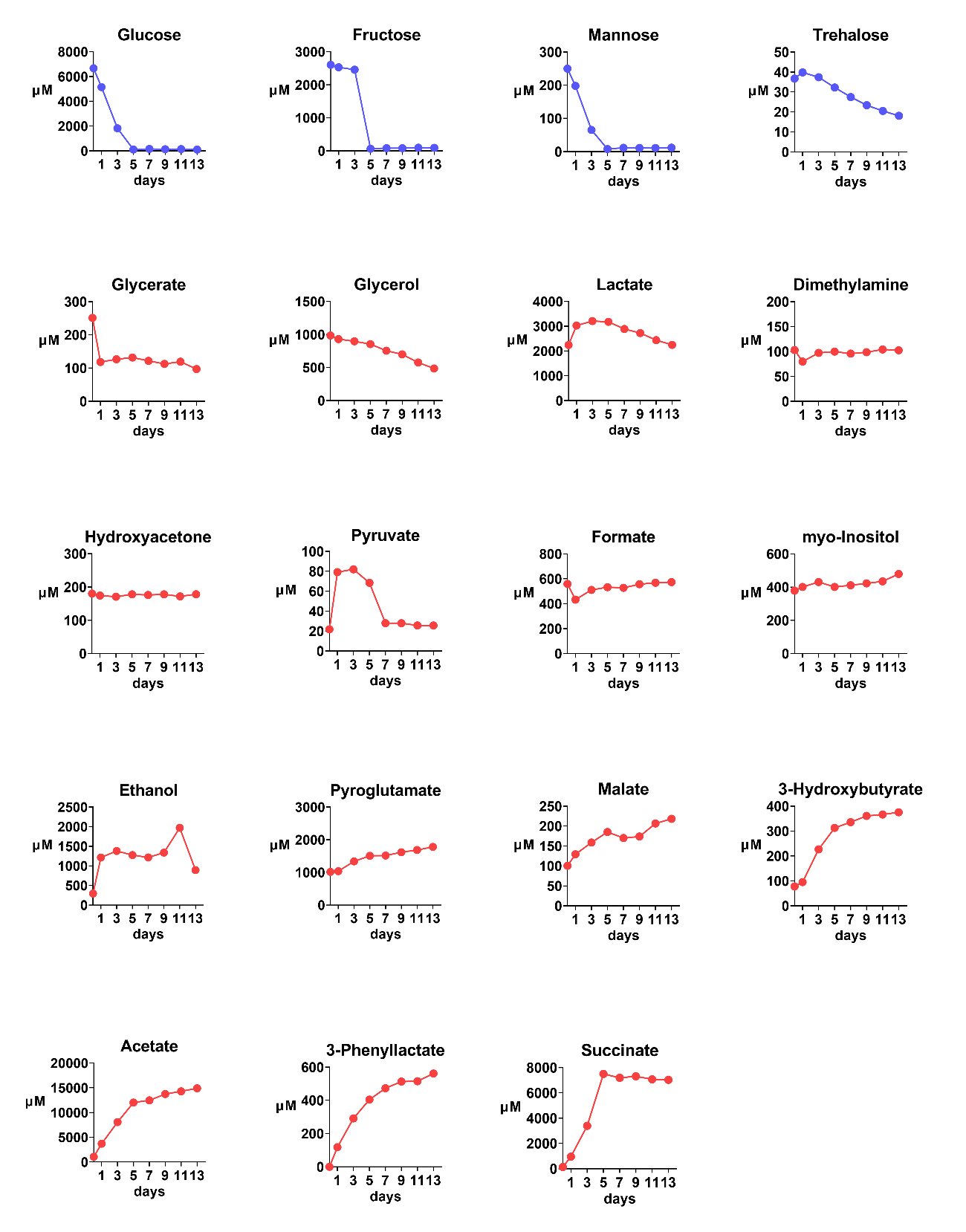


Continuation of Fig. S2


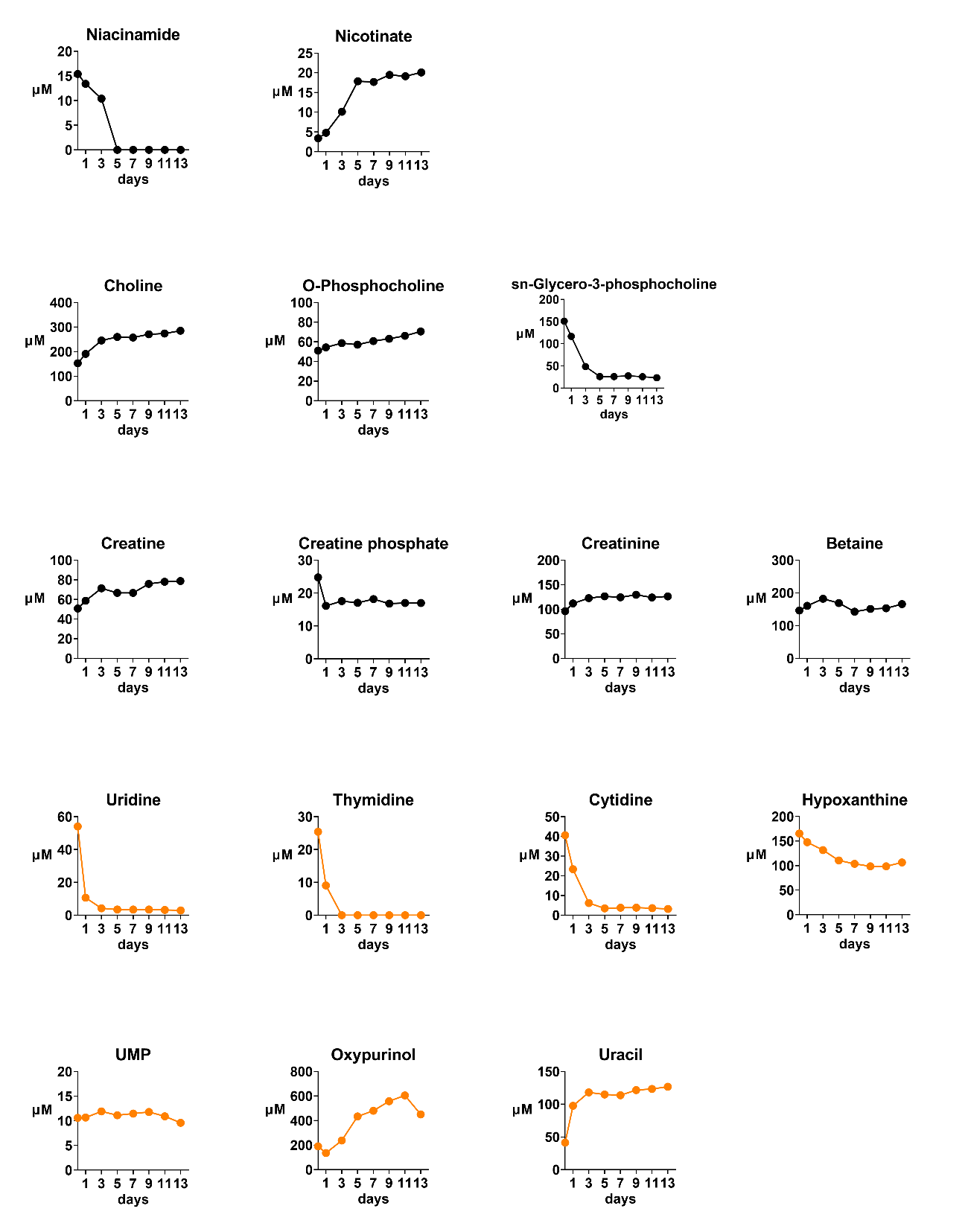


**Fig.** S3 Alignment of SDR-TDH sequences. Residues from TcTDH involved in potassium coordination are highlighted in red. Residues mutated to alanine for kinetic studies are labeled with a star.


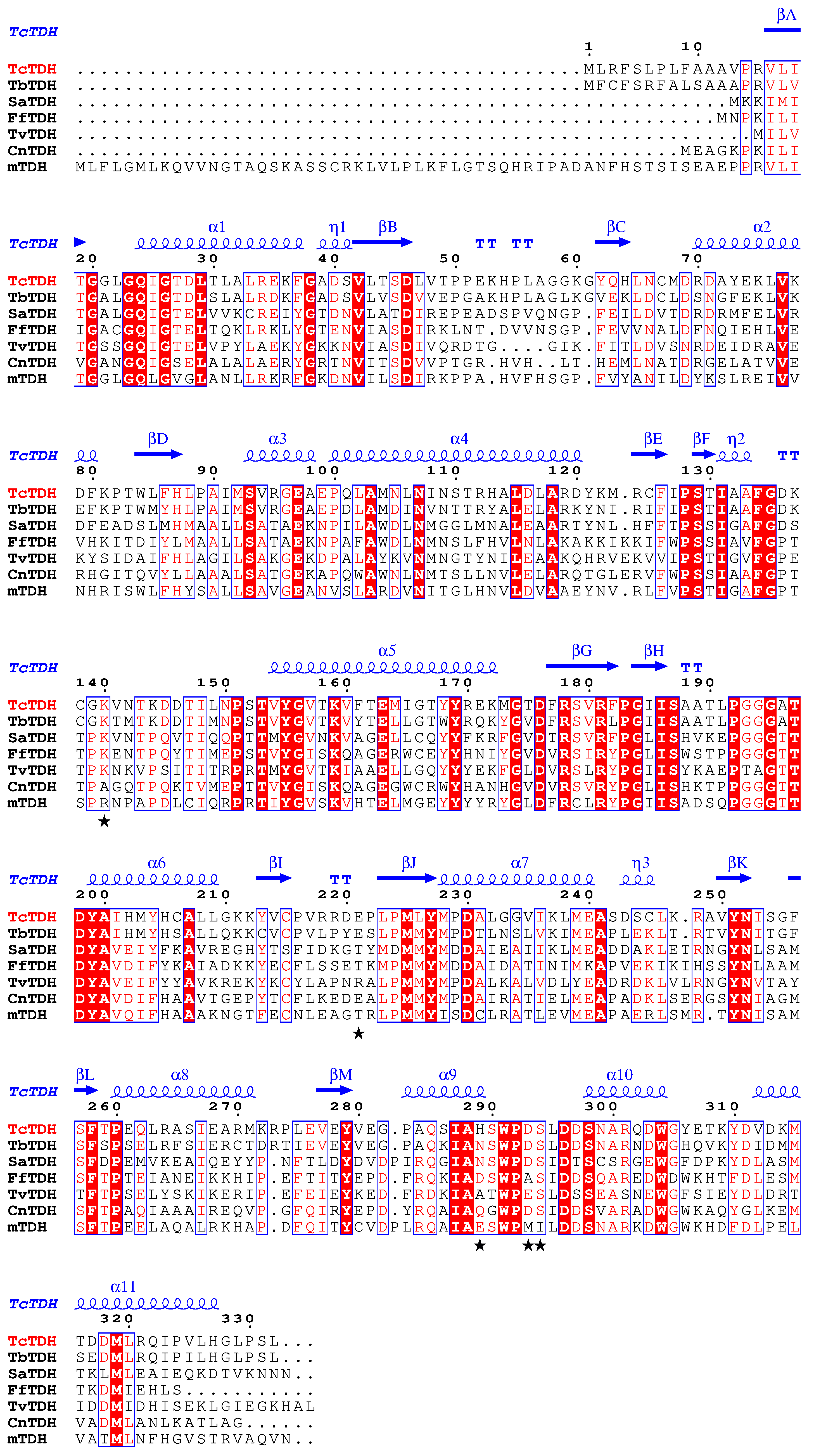


***Trypanosoma cruzi* strain identification**

To identify the *T. cruzi* strain used in this project, an 832 bp fragment of the highly polymorphic *T. cruzi* gene encoding the lathosterol/episterol oxidase enzyme (TcSC5D), involved in the sterol biosynthesis pathway, was amplified by PCR and sequenced, following the protocol adapted from Consentino & Agüero (2012). In brief, Genomic DNA was extracted from *T. cruzi* using the Wizard® Genomic DNA Purification Kit (Promega, Madison, WI, USA) according to the manufacturer’s protocol. PCR amplification of the *TcSC5D* gene was performed using the oligonucleotide primers: *TcSC5D*.For: 5’ GGACGTGGCGTTTGATTTAT 3’ and *TcSC5D*.Rev: 5’ TCCCATCTTCTTCGTTGACT 3’. The reaction was carried out with Phusion High-Fidelity DNA Polymerase Master Mix (Thermo Fisher Scientific, Waltham, MA, USA) on a GeneAmp PCR System 9700 Thermal Cycler (Applied Biosystems, Waltham, MA, USA). PCR conditions consisted of an initial denaturation at 94 °C for 5 minutes, followed by 35 cycles of denaturation at 94 °C for 30 seconds, annealing at 57 °C for 30 seconds, and extension at 72 °C for 1 minute, with a final extension step at 72 °C for 5 minutes. The amplified PCR products were resolved on a 0.8% agarose gel and purified using the Gel & PCR Purification Kit (Cellco Biotec, São Carlos, SP, Brazil). The purified PCR product was subsequently subjected to Sanger sequencing. The resulting *TcSC5D* sequence was compared against all available *T. cruzi* genomes in TriTrypDB database. Blast analysis indicated the strain to be Dm28c. *TcSC5D* sequenced from the strain used in this study (*TcSC5D*_Seq) and the gene with 100% identity found in TriTrypDB database from Dm28c (*BCY84_02439*_Dm28c) were aligned in Clustal Omega (EMBL-EBI) (Fig. S4). Besides, single nucleotide polymorphisms (SNPs) identified in the sequence were compared to the SNPs described by Consentino & Agüero (PLoS Negl Trop Dis, 2012; 6(7):e1777) for lineage/discrete typing units (DTU) differentiation and matched with DTU Tc-I (Fig. S4).

**Fig.** S4 Alignment of TcSC5D sequenced from the strain used in this study (TcSC5D_Seq) and strain Dm28c (gene BCY84_02439) using Clustal Omega (EMBL-EBI). Only the sequencing region with a high degree of reliability was utilized for the identification of the *T. cruzi* strain. Consequently, two regions of 38 bp each, located at the beginning and the end of the *TcSC5D*, were excluded from the analysis. The SNPs for lineage/discrete typing units (DTU) differentiation are highlighted in red. All SNPs in the *TcSC5D* correspond to the ones described for *T. cruzi* DTU-TcI .

*TcSC5D*_Seq TGTAGATCGTGACGCGTTATCTCATCAATTATTTGTTTTTTGGTTAATTCTTACGACTGG 60

*BCY84_02439*_Dm28c TGTAGATCGTGACGCGTTATCTCATCAATTATTTGTTTTTTGGTTAATTCTTACGACTGG 60

************************************************************

*TcSC5D*_Seq TGGTATATTTTTATACCTTTTATGCGCATCCATTTCAACATTTATCTTTTTTGTGTTATT 120

*BCY84_02439*_Dm28c TGGTATATTTTTATACCTTTTATGCGCATCCATTTCAACATTTATCTTTTTTGTGTTATT 120

************************************************************

*TcSC5D*_Seq TGAGGAAACTTATTTTCCTCACACGATGGATAAAAAAAATCAAAAACATGAATTACAACG 180

*BCY84_02439*_Dm28c TGAGGAAACTTATTTTCCTCACACGATGGATAAAAAAAATCAAAAACATGAATTACAACG 180

************************************************************

*TcSC5D*_Seq ACAAATGTTACATGAGATATTTATTGCGGTTCTTTCTATTCCTTTTATGGCAATATTAAT 240

*BCY84_02439*_Dm28c ACAAATGTTACATGAGATATTTATTGCGGTTCTTTCTATTCCTTTTATGGCAATATTAAT 240

************************************************************

*TcSC5D*_Seq GGCCCCTTCTTCTACCCTTGCCTACCGTGGGTACAGTAAAATATATTACAATGTTTCCGA 300

*BCY84_02439*_Dm28c GGCCCCTTCTTCTACCCTTGCCTACCGTGGGTACAGTAAAATATATTACAATGTTTCCGA 300

************************************************************

*TcSC5D*_Seq TTATGGATGGTCATATCTTTTTTTAAGTATTTTGATGTTTTTTATCTTTACGGATTTTAT 360

*BCY84_02439*_Dm28c TTATGGATGGTCATATCTTTTTTTAAGTATTTTGATGTTTTTTATCTTTACGGATTTTAT 360

************************************************************

*TcSC5D*_Seq GGTTTATTGGTTTCATCGTGGTTTACATCATCCCACATTATACCGATATCTTCATAAATT 420

*BCY84_02439*_Dm28c GGTTTATTGGTTTCATCGTGGTTTACATCATCCCACATTATACCGATATCTTCATAAATT 420

************************************************************

*TcSC5D*_Seq ACATCATACATACAAATATACCACACCATTTTCATCTCATGCATTTAATCCTTGTGATGG 480

*BCY84_02439*_Dm28c ACATCATACATACAAATATACCACACCATTTTCATCTCATGCATTTAATCCTTGTGATGG 480

************************************************************

*TcSC5D*_Seq ATTTGGTCAAGGTTCACCATATTATGCATTTATTTTTTTATTTCCTATGCATAATTATCT 540

*BCY84_02439*_Dm28c ATTTGGTCAAGGTTCACCATATTATGCATTTATTTTTTTATTTCCTATGCATAATTATCT 540

************************************************************

*TcSC5D*_Seq TTTTGTTATTCTCTTTTTTGCCGTCAATTTATGGACCATCTCCATTCACGATCAGGTGGA 600

*BCY84_02439*_Dm28c TTTTGTTATTCTCTTTTTTGCCGTCAATTTATGGACCATCTCCATTCACGATCAGGTGGA 600

************************************************************

*TcSC5D*_Seq TTTTGGGGGGCATTTTGTTAACACAACCGGGCATCATACAATTCATCATGTACTTTTTAA 660

*BCY84_02439*_Dm28c TTTTGGGGGGCATTTTGTTAACACAACCGGGCATCATACAATTCATCATGTACTTTTTAA 660

************************************************************

*TcSC5D*_Seq TTACGACTACGGACAATACTTCACCGTATGGGATCGTATTGGTGGAACGTATAAACCGGC 720

*BCY84_02439*_Dm28c TTACGACTACGGACAATACTTCACCGTATGGGATCGTATTGGTGGAACGTATAAACCGGC 720

************************************************************

*TcSC5D*_Seq ACAACAGACGCATCATTTCCCGTTATTTACAAAA 754

*BCY84_02439*_Dm28c ACAACAGACGCATCATTTCCCGTTATTTACAAAA 754

**********************************

**Table S1.** Crystallography statistics.

| **Data Collection Statistics** | **holo-TcTDH** | **apo-TcTDH** |
| --- | --- | --- |
| Resolution (Å) | 47.27-1.73 (1.77-1.73) | 46.76-2.10 (2.16-2.10) |
| Space group | P12_1_1 | P12_1_1 |
| Cell Parameters |  |  |
| *a*; *b*; *c* (Å) | 47.27; 82.73; 84.38 | 46.69; 81.83; 83.67 |
| *α* = *γ ≠ β* (°) | 90; 90.45 | 90; 90.55 |
| No. of unique reflections | 67,241 (3,667) | 36,424 (2,927) |
| Rmeas§ | 0.085 (0.416) | 0.156 (1.03) |
| Completeness (%) | 99.8 (99.3) | 99.1 (98.6) |
| <I/σ(I)> | 7.8 (0.7) | 8.4 (1.8) |
| CC1/2 | 0.997 (0.466) | 0.995 (0.595) |
| Multiplicity | 2 (2) | 4.7 (4.5) |
| Wilson B factor (Å2) | 22.87 | 27.5 |
| **Refinement Statistics** |  |  |
| *R*work | 0.18 | 0.20 |
| *R*free | 0.21 | 0.25 |
| Amino acid residues | 637 | 638 |
| Heteroatoms(n) | 124 | 2 |
| Solvent (n) | 285 | 86 |
| Average B factor (Å2) |  |  |
| Protein | 24.9 | 34.7 |
| NAD^+^ | 18.7 | - |
| Acetate | 24.5 | - |
| Ion potassium | 41.0 | 70.3 |
| Bis-Tris | 29.3 | - |
| Glycerol | 52.0 | - |
| Water | 32.4 | 30.6 |
| RSCC/RSR |  |  |
| NAD^+^ | 0.99/0.07 | - |
| Acetate | 0.97/0.08 | - |
| Ion potassium | 0.98/0.13 | 0.96/0.19 |
| Bis-Tris | 0.96/0.19 | - |
| Glycerol | 0.86/0.14 | - |
| **Validation** |  |  |
| Ramachandran |  |  |
| Favored | 615 (97%) | 610 (96%) |
| Allowed | 12 (2%) | 22 (3.5%) |
| PDB ID | 8GJB | 8GIL |

§ Rmeas = Σ*hkl* √[N/(N-1)] Σ*i* |*Ii*(*hkl*) - ‹*I*(*hkl*)› | / Σ*hkl* Σ*i* *Ii*(*hkl*)

**Table S2.** Oligonucleotides used for site-directed mutagenesis of TcTDH**.**

| **Mutant** | **Sense** | **Sequence (5’ → 3’)** |
| --- | --- | --- |
| D293A | Fwd  Rev | AGCTGGCCGGCCAGCCTGGAC  GTGCGCAATGCTCTGCGC |
| E221A | Fwd  Rev | GGTGCGTCGTGATGCGCCGCTGCCGATG  CATCGGCAGCGGCGCATCACGACGCACC |
| H289A | Fwd  Rev | GAGCATTGCGGCCAGCTGGCCGGACAGC  TGCGCCGGACCCTCAACA |
| K140A | Fwd  Rev | CAAATGCGGCGCGGTTAACACCAAGGACGATAC  TCGCCAAACGCCGCAATG |
| S294A | Fwd  Rev | CTGGCCGGACGCCCTGGACGATAGC  CTGTGCGCAATGCTCTGC |
